# Frozen Funding on Firearm Research: “Doing Nothing Is No Longer an Acceptable Solution”

**DOI:** 10.5811/westjem.2016.1.29767

**Published:** 2016-01-14

**Authors:** Marian E. Betz, Megan L. Ranney, Garen J. Wintemute

**Affiliations:** * University of Colorado School of Medicine, Department of Emergency Medicine, Denver, Colorado; † Rhode Island Hospital/Alpert Medical School, Brown University, Department of Emergency Medicine, Providence, Rhode Island; ‡ University of California, Davis, Violence Prevention Research Program, Department of Emergency Medicine; Sacramento, California

December 2015 saw another Congressional budget standoff and threatened government shutdown. This omnibus bill was particularly important for public health, because – for the first time in years – it contained language that would have reversed a 19-year-old prohibition on Centers for Disease Control and Prevention (CDC) funding for research on firearm injury. Unfortunately, 2016’s final Omnibus Appropriations bill did not reverse this prohibition. And so another year begins with the United States – and the world – debating how to solve the problem of firearm violence in this country, without the benefit of objective public health research.

## What is the Funding “Ban”?

The so-called funding ban began in 1996, when Representative Jay Dickey, a junior Republican from Arkansas, inserted a rider into the federal spending bill, with support and lobbying from the National Rifle Association. The rider stipulated that no CDC funds “may be used to advocate or promote gun control.” Congress also cut CDC’s budget by exactly the amount dedicated to firearm injury research. Although the amendment did not prohibit research per se, it was interpreted as doing so. As such, it has had a chilling effect on research, effectively halting federally-funded research on firearm injury and freezing the number of publications.^1^,^2^ By 2011, similar language had been extended to cover the National Institutes of Health (NIH).

In January 2013, in response to the shooting at Sandy Hook Elementary School in Newtown, Connecticut, President Obama released a plan to reduce gun violence. This plan included a presidential memorandum clarifying the meaning of the Dickey amendment and directing the CDC and other scientific agencies within the Department of Health and Human Services (including the NIH) to “conduct or sponsor research into the causes of gun violence and the ways to prevent it.”^3^ Since then, the NIH has funded three studies explicitly related to firearm injury,^4^ and the National Institute of Justice awarded nearly $2 million in funding for firearms-related research in 2014.^5^ Yet Congress has continued to quietly – and sometimes not so quietly – block additional funds for firearm research and restrict the CDC’s budget to limit activities related to firearm violence.

Though his amendment remains in force, Rep. Dickey’s views have changed. In December 2015, he published an open letter to Congress urging action on firearm injuries and lamenting the way his intent (to avoid federal funding for politicized gun control advocacy) was distorted to obstruct scientific and public health progress. “Research could have been continued on gun violence without infringing on the rights of gun owners, in the same fashion that the highway industry continued its research without eliminating the automobile,” he wrote. “It is my position that somehow or someway we should slowly but methodically fund such research until a solution is reached. Doing nothing is no longer an acceptable solution.”^6^

Unfortunately, in the face of the 2016 Omnibus Appropriations bill, we are being asked to continue to “do nothing” in the research realm.

## What Does This “Ban” Mean for Emergency Physicians?

In emergency medicine, we deal with firearm injuries and their effects on a daily basis. With our pre-hospital and surgical colleagues, we treat the victims of acute violence (be it from domestic violence, other assaults, mass shootings, or self-inflicted wounds) and survivors suffering from chronic physical and mental sequelae of firearm injuries.

We know that comprehensive research strategies have dramatically decreased total motor vehicle deaths and age-adjusted death rates over the past three decades, even after accounting for increases in road travel (Figure). Had we, as Rep. Dickey and others have suggested,^1^,^7^,^8^ applied similar strategies from public health and medical research to firearm injury, we might be in a very different spot today. But because we lack critical information on the epidemiology of firearm violence and on the effectiveness of various strategies to prevent it, the number and rates of firearm deaths have remained steady.

While we know how to resuscitate a patient with a gunshot wound, prevention matters, too. Many who die from firearm injuries never make it to through our emergency departments’ doors; this is especially true for firearm suicides, which have a case fatality rate of 85%.^9^ And triage and prevention is part and parcel of our specialty: we are expected to first predict who is at high risk for myocardial infarction, arrhythmia, suicide, and stroke, and then to intervene appropriately. For firearm injury, we are largely at a loss. Which patients are at highest risk for injury? How can we prevent future firearm injury for the adolescent in a gang, the woman in an abusive relationship, or the depressed older man? How should we counsel our patients about firearm storage or ownership? Which policies should we, as medical professionals, advocate for as evidence-based solutions? The CDC’s website contains myriad fact sheets and reports about motor vehicle safety, but almost nothing about firearms. For example, the online CDC A–Z Index “lists topics with relevance to a broad cross-section of CDC.gov’s audiences…. The items are representative of popular topics, frequent inquiries, or have critical importance to CDC’s public health mission.”^10^ There are over 2,300 topics listed in the index, ranging from “abrasive blasting” to “Zika virus,” topics that each have a whole webpage and fact sheets. “Fireworks” and “nail guns” each have an entry, as does “motor vehicle injuries,” but both “firearm” and “gun” are missing completely.

## So What Can Emergency Physicians *Do?*

The scarcity of federal funding for firearm research may slow us down, but we cannot allow it to stop the quest for truth and improving the public’s health. Rather than throwing up our collective hands, we can and should take steps –even if they’re small steps– forward. We can do so in four ways.

First, we need to engage junior investigators in firearm-related research. The dearth of funding has not only stopped projects but has also dried up the pipeline of scientists entering the field. Only a handful of experienced investigators in firearm violence are actively working in the United States. It is critically important for junior investigators to work with these senior investigators; opportunities for collaboration can include writing, presentations, and participation in workgroups or research projects.

Second, recognizing that funding is the sine qua non of high-quality research, we must pursue alternate sources of funding while federal funding lags. For instance, some researchers have successfully obtained foundation money to conduct firearm injury prevention research. Engaging foundations in our research enterprise is critical for developing a new research workforce and trustworthy outcomes. Yet we must also continue to apply for federal funding, including from the NIH, to enhance the scope and prominence of projects. It is key to remember that no actual “ban” exists and federal funding is possible, albeit inadequate and difficult to obtain.

Third, we need continued leadership from emergency medicine organizations.^11^,^12^ The American College of Emergency Physicians (ACEP) recently convened an expert group to develop a prioritized research agenda related to firearm violence, due to be published this year. ACEP also publicly called for more federally-funded firearm injury research, joined by six physician professional societies (American Academy of Family Physicians, American Academy of Pediatrics, American Congress of Obstetricians and Gynecologists, American College of Physicians, American College of Surgeons, and American Psychiatric Association), the American Public Health Association, and American Bar Association.^13^ These organizations, with the help of their members, can also encourage expansion of the national surveillance systems – such as the National Violent Death Reporting System, which collects epidemiologic data on violent deaths – with which more rigorous injury prevention research could be conducted.^14^

Finally, we need to use respectful conversation and collaboration to help bridge the political chasm over firearm injury prevention.^15^ We should not assume that responsible gun owners are opposed to research aimed at reducing firearm injury, nor should we assume that the National Rifle Association accurately represents the views of most gun-owning Americans. Similarly, we should not assume that non-gun owners are trying to confiscate guns or otherwise infringe on Constitutional rights. An estimated 41% of emergency physicians own a firearm, based on a 2011–2012 survey of a sample of ACEP members.^16^ Leadership from gun owning physicians in calling for more funding and research in firearm injury prevention could be particularly powerful.

In conclusion, we need research to know what works to prevent firearm injury, when, with whom, and how. We must not lose sight of the larger goal of increasing federal support for firearm violence research, but we also must not lose time waiting for the money to appear before we take action. Our patients and our communities deserve safety.

## Figures and Tables

**Figure:**
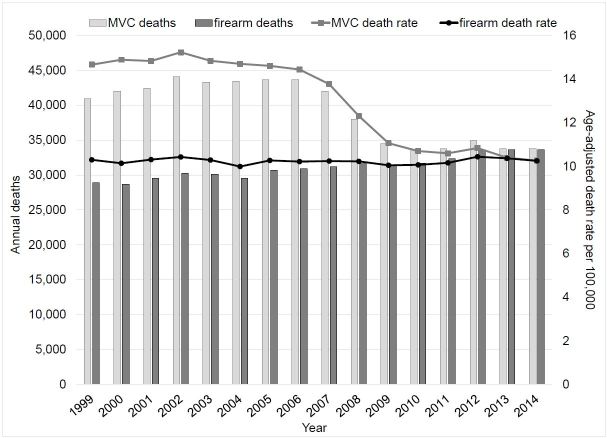
Firearm and motor vehicle collision (MVC) deaths (US, 1999–2014).^17^
